# Hepatitis B virus‐induced hyperactivation of B cells in chronic hepatitis B patients via TLR4

**DOI:** 10.1111/jcmm.15202

**Published:** 2020-05-11

**Authors:** Yang Li, Shengxia Yin, Yuxin Chen, Quan Zhang, Rui Huang, Bei Jia, Wei Jie, Kefang Yao, Jian Wang, Xin Tong, Yong Liu, Chao Wu

**Affiliations:** ^1^ Department of Infectious Diseases Nanjing Drum Tower Hospital Nanjing University Medical School Nanjing China; ^2^ Department of Laboratory Medicine Nanjing Drum Tower Hospital Nanjing University Medical School Nanjing China; ^3^ Department of Experimental Medicine Nanjing Drum Tower Hospital Nanjing University Medical School Nanjing China

**Keywords:** B cell hyperactivation, chronic hepatitis B, NF‐κB pathway, TLR4

## Abstract

B cell hyperactivation and functional impairment were identified from patients with chronic hepatitis B virus (CHB) infection; however, the underlying mechanism remains unknown. Here, we aim to elucidate the mechanisms responsible for B cell hyperactivation during HBV infection. Peripheral CD19^+^ B cells isolated from 4 CHB patients and 4 healthy volunteers were analysed by RNA sequencing. A total of 1401 differentially expressed genes were identified from B cell transcriptome of CHB patients vs healthy volunteers. We found that B cells from CHB patients were functional impaired, with increased TLR4 expression, activated NF‐κB pathway and altered mitochondrial function. The expression of B cell activation‐related genes, including TLR4, was further validated using additional clinical samples. To further verify the role of TLR4 in B cell activation during CHB, B cell phenotypes were determined in wild‐type (WT) and TLR4^−/−^ HBV‐carrier mice. Hyperactivated B cell and TLR4 signalling pathway were observed in WT HBV‐carrier mice, while TLR4 ablation failed to induce B cell hyperactivation, and downstream MyD88 and NF‐κB were also not altered. Taken together, TLR4 pathway plays a pivotal role in B cell hyperactivation during CHB, which might serve as a promising target for B cell function restoration.

## 1 INTRODUCTION

Chronic hepatitis B virus (CHB) infection remains to pose a global public health challenge, affecting a population of 240 million people worldwide.^1^ Persistent hepatitis B virus (HBV) infection may result in progressive liver disease that leads to cirrhosis, hepatic failure and hepatocellular carcinoma (HCC).^2^ Despite current antiviral therapies have much improved outcome, few patients achieve the ultimate goal of hepatitis B surface antigen (HBsAg) loss and anti‐HBs antibody seroconversion.^3^ Recent evidence has highlighted an essential role of HBV‐specific B cells in effective control of CHB infection.^4^, ^5^, ^6^ Nevertheless, our recent work has shown that the number of HBsAb‐secreting B cells was remarkably reduced in CHB patients.^7^

Chronic microbial infections are frequently associated with B cell activation and aberrant polyclonal proliferation, which might further impede the development of pathogen‐specific humoral response. Previous studies have revealed the activation and impaired functional capacity of the global B cell population typically observed in CHB patients.^8^, ^9^ Further, we also showed that B cell hyperactivation and the serological IgG levels were significantly higher in CHB patients, while decreased HBsAg‐specific B‐cell responses were associated with HBV persistence in CHB patients.^7^, ^10^ Additionally, our work also suggested a significant elevation of B cell subpopulation, regulatory B cells (Bregs), which could modulate immune responses during CHB progression.^11^ All these studies suggest that HBV is able to induced B cell hyperactivation and functional impairment. Nevertheless, the underlying mechanisms responsible for B cell hyperactivation and aberrant proliferation in CHB are not fully understood.

In this study, with the intent of gaining important insight of B cell pathobiology in CHB, we perform a genome‐wide expression profile of B cells from CHB patients, which revealed an extensively significant distinct gene expression signature when compared to functionally competent B cells from healthy volunteers. Of note, the significant up‐regulation of TLR4 and myeloid differentiation factor 88 (MyD88) gene was unearthed and pathway analysis showed that nuclear factor κ‐light‐chain‐enhancer of the activated B cell (NF‐κB) pathway was enriched. We hypothesize that the activation of TLR4/MyD88/NF‐κB pathway triggered by HBV infection may be involved in B cell proliferation and activation among CHB patients. To further validate our assumption, HBV‐carrier mouse model was used to dissect the role of TLR4 and downstream signalling pathway on B cell proliferation and activation in the context of CHB. Our data indicate that TLR4 is required for B cell hyperactivation during CHB via activating the downstream MyD88/ NF‐κB signalling pathway. These results identify TLR4 as a promising target to reduce B cell hyperactivation and aberrant proliferation in CHB.

## 2 MATERIALS AND METHODS

### 2.1 Study subjects

CHB patients of immune active HBeAg‐positive (IA) were enrolled into the study from the Outpatient Hepatitis Clinic at Nanjing Drum Tower Hospital. Following on 2012 clinical guidelines for chronic hepatitis B prepared by the European Association for the Study of the Liver (EASL),^12^ CHB IA patients were ‘immune reactive HBeAg‐positive phase’, defined as relatively lower level of HBV DNA levels (>2000 IU/mL) compared with the immune tolerant phase, increased or fluctuating levels of aminotransferases, moderate or severe liver necroinflammation and more rapid progression of fibrosis. These CHB IA patients were positive HBsAg for at least 6 months and had not been previously treated with a history of interferon (IFN) or nucleos(t)ide analogues (NUC) therapy. Age‐ and sex‐matched healthy volunteers previously immunized with hepatitis B vaccine were also included as controls.

All the subjects included were negative for antibodies against other types of hepatitis or human immunodeficiency virus. Meanwhile, patients with liver cirrhosis, hepatocellular carcinoma, hypertension, diabetes, heart disease and other major diseases were excluded. All the subjects provided informed consents, and this study was approved by the institutional review boards (IRB) of Nanjing Drum Tower Hospital, in accordance with the Helsinki Declaration and guidelines of the Nation Health and Medical Research Council of China.

### 2.2 Cell isolation and RNA‐Seq

The peripheral blood mononuclear cells (PBMCs) were freshly isolated from venous blood of 4 immune‐activated CHB patients with positive HBeAg and 4 healthy controls with the prior history of HBV vaccine immunization. Heparinized blood mixed with two volumes of PBS (pH 7.4) was subjected to Lymphoprep^TM^ (Stem Cells) density‐gradient centrifugation. B cells were purified by negative selection using a Human B Lymphocyte Enrichment Set (BD Biosciences), in which the Biotinylated Human Enrichment Cocktail with the following antibodies Anti‐CD3, Anti‐CD41α, Anti‐CD43 and Anti‐CD235α recognizes antigens expressed on erythrocytes, platelets and peripheral leukocytes, but not B lymphocytes.

Total RNA was isolated from the purified B cell population by RNeasy Mini Kit (Qiagen). The cDNA libraries were generated using the VAHTS mRNA‐Seq v2 Library Prep Kit for Illumina^®^ (Vazyme) following the manufacturer's instruction. First, mRNA was purified from total RNA using poly‐T oligo‐attached magnetic beads. Fragmentation was performed using divalent cations under elevated temperature in Vazyme Frag/Prime Buffer. The cleaved RNA fragments were copied into first‐strand cDNA using reverse transcriptase and random primers. Second‐strand cDNA synthesis was subsequently performed using buffer, dNTPs, DNA polymerase I and RNase H. cDNA fragments were end‐repaired with the addition of a single ‘A’ base at the 3'‐end of each strand, subsequently ligated with the special sequencing adapters (Vazyme). The products were purified and size selected with VAHTSTM DNA Clean Beads (Vazyme) for sequencing.

Then, cDNA library was quantified using Qubit^®^ RNA Assay Kit via Qubit^®^3.0 (Life Technologies). The clustering of the index‐coded samples was performed on a cBot Cluster Generation System (Illumina) according to the manufacturer's instruction. After cluster generation, the library preparations were sequenced on a Hiseq X Ten platform (Illumina).

### 2.3 Data analysis of RNA‐Seq

All gene‐level read counts were imported into Agilent GeneSpring GX software version 11.5.1 (Agilent Technologies) for further analysis. To evaluate biological significance of the changes among genes, network analysis of the differentially expressed genes (DEGs) was performed, using the CapitalBio‐Molecule Annotation System (MAS) software (http://bioinfo.capitalbio.com/mas3/). DEGs are defined as a gene with a fold change ≥ 1.5 and a *P* value ≤ .05. Hierarchical clustering and principal components analysis using an uncentred correlation distance metric and average linkage clustering were performed in Cluster with visualization in TreeView (http://www.treeview.net). *P* Values used in the pathway and Gene Ontology (GO) analysis were calculated according to hypergeometric distribution probability formula. The *P* value or *q* value reflects the importance of the pathway or GO. To determine the most significant biological functions and pathways of the DEGs, three major annotation databases including GO, Kyoto Encyclopedia of Genes and Genomes (KEGG) and Reactome were applied in the present study.

### 2.4 HBV‐carrier mouse model and isolation of mouse B cells

C57BL/10 mice and TLR4^−/−^ mice (male, 6‐8 weeks old) were purchased from Nanjing Biomedical Research Institute of Nanjing University. Mice were housed at SPF Animal Center of Nanjing Drum Tower Hospital. All mouse experiments were approved by Institutional Animal Care and Use Committee (IACUC) at Nanjing Drum Tower Hospital. The hydrodynamic injection (HDI)‐based HBV‐carrier models were generated as previously described by using pAAV‐HBV1.2 plasmid,^13^ which was kindly provided by Dr Pei‐Jer Chen (National Taiwan University College of Medicine). pAAV empty plasmid was used for control group. The plasmids were isolated by using an endotoxin‐free Maxi kit (Qiagen). Briefly, 8 μg of the pAAV/HBV1.2 or pAAV plasmid was prepared in 2 mL saline and injected via tail veil within 10 seconds. Mouse mononuclear cells were isolated from liver, spleen and bone marrow by density gradient centrifugation using a percoll cushion. Mouse B cells were purified by negative selection using Mouse B Lymphocyte Enrichment Set (BD Bioscience). Serum IgG levels in C57BL/10 mice and TLR4^−/−^ mice were measured with enzyme‐linked immunosorbent assay kits (ELISA, Lianke bio) according to the manufacturer's instructions.

### 2.5 Quantitative real‐time RT‐PCR

Total RNA from lysed cells was extracted from the purified B cells with the RNeasy Mini Kit (Qiagen) according to the manufacturer's instructions. Reverse transcription was conducted using Superscript II Reverse Transcriptase (TAKARA Bio) with random hexamer primer and oligo‐dT. Real‐time RT‐PCR was performed using commercially available TaqMan gene expression probes (Applied Biosystems) for human B cell–related genes, including *bach2*, *id3*, *cd69*, *xbp1*, *irf4*, *prdm1*, *ctla4*, *id3*, *tlr4*, *nfam1*, *peli1*, *zap70*, *stk39*, *nod2* and *gapdh*. The expression level of each gene of interest was normalized to GAPDH, and the results were given as relative copy numbers.

Gene expression in mouse B cells including TLR4, Myd88, Bach2, NFAM1 and CTLA4 was determined by real‐time RT‐PCR using SYBR‐Green qPCR Master Mixes (Thermo Fisher Scientific). The relative fold change was calculated based on the 2^−ΔΔ^ *^C^* ^t^ method. The relative quantity of the target mRNA was normalized to the level of β‐actin mRNA.

### 2.6 Flow cytometry for B cell subset phenotype

All mAbs were purchased from BD Biosciences (BD Pharmingen™). PBMC was isolated from immune‐activated CHB patients with positive HBeAg and healthy controls and stained with the following mAbs: anti‐CD19‐PE‐CY7, anti‐CD27‐FITC, anti‐CD69‐PerCP, anti‐CD80‐PE, anti‐CD86‐APC or anti‐TLR4‐PE. For mouse B cells, the expression of CD27, CD45, CD80, CD86 and TLR4 on B cells was also detected by flow cytometric analysis on BD FACS Aria II (BD Biosciences). The flow cytometry analysis was performed on BD FACS Aria II using FACSDiva software (BD Biosciences). Appropriate isotype controls were performed in parallel at the saturating concentrations.

### 2.7 Western blot analysis

Protein lysates were prepared from purified mouse B cells with RIPA lysis buffer supplemented with protease inhibitor cocktail (Sigma). The protein expression levels of MyD88 and NF‐κB p65 protein were detected by Western blotting. Briefly, equal amounts of protein were electrophoresed on an SDS‐PAGE then transferred onto a nitrocellulose membrane, which was blocked in Tris‐buffered saline with Tween (TBST) containing 5% non‐fat dry milk. Then, the membranes were incubated with rabbit polyclonal MyD88 antibody (Abcam), rabbit monoclonal NF‐κB p65 antibody (Abcam) and rabbit polyclonal β‐actin antibody (Abcam), respectively. Blots were visualized after incubation with goat horseradish peroxidase (HRP)‐conjugated IgG antibodies. Signals were detected using enhanced chemiluminescence (Bio‐Rad).

### 2.8 Statistical analysis

Statistical analyses were performed using SPSS 21.0 (IBM‐SPSS Inc). Statistical analysis was performed with Pearson's chi‐square test, Student's *t* test and Mann‐Whitney *U* test where appropriate. All estimates accompanied by two‐sided *P* values of <.05 were considered statistically significant.

## 3 RESULTS

### 3.1 3.1Genome‐wide expression profiles of B cells between CHB patients and HBV vaccinated healthy controls

**Figure 1:**
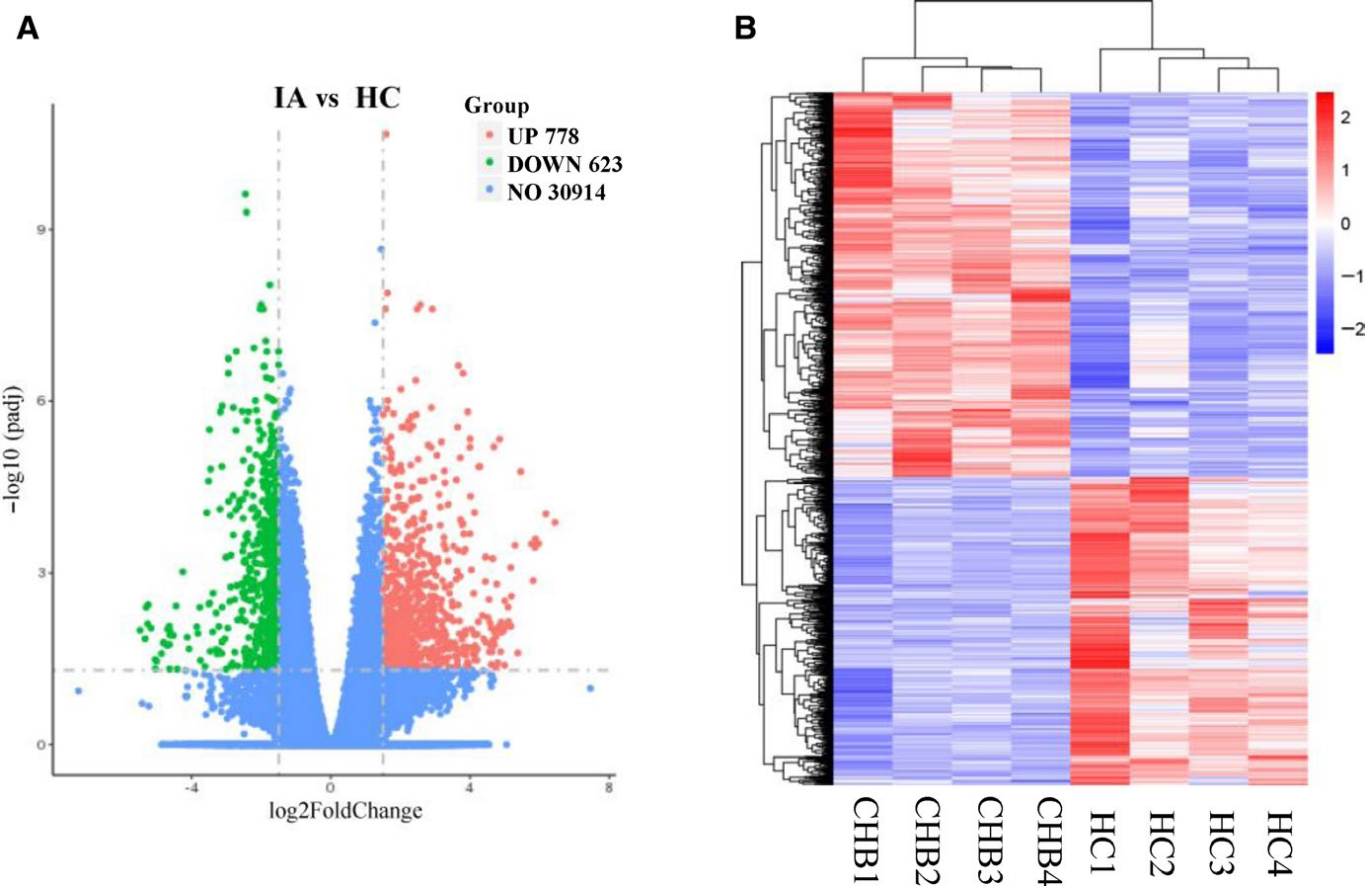
Genome‐wide expression profiles of B cells between CHB patients and healthy controls. A, Volcano map of differentially expressed B cell genes. Significantly differentially expressed genes (DEGs) are shown as a red (up) or green (down) dot. Genes without significant changes are shown as blue dot. B, Heatmap of gene expression values depicting gene clusters in each B cell sample. Sample names are represented in columns, and significant genes are represented in rows. Genes and samples are clustered together based on expression similarity. Low to high expression is represented by a change of colour from blue to red, respectively

In order to investigate any differences in gene expression profiles of B cells between CHB patients and healthy subjects, RNA‐sequence analysis of B cells was conducted in 4 CHB patients and 4 HBV vaccinated healthy subjects (Table S1), in which a total of 32 315 genes were detected for the reads when aligned to human genome. The results are shown in a volcano plot (Figure 1A). Hierarchical cluster analysis was conducted for changed genes with a fold change > 1.5 (*P* < .05, FDR < 30%) or a fold change < 0.667 (Figure 1B). Expectedly, the 8 samples were categorized into two main distinct clusters, and a subset of 1401 genes were significantly differentially expressed in the CHB groups compared with the healthy groups. Of these, 778 genes were up‐regulated, whereas 623 genes were down‐regulated (Figure 1A).

### 3.2 3.2Gene‐set enrichment analysis (GSEA) for DEGs in CHB patients and healthy control

**Figure 2:**
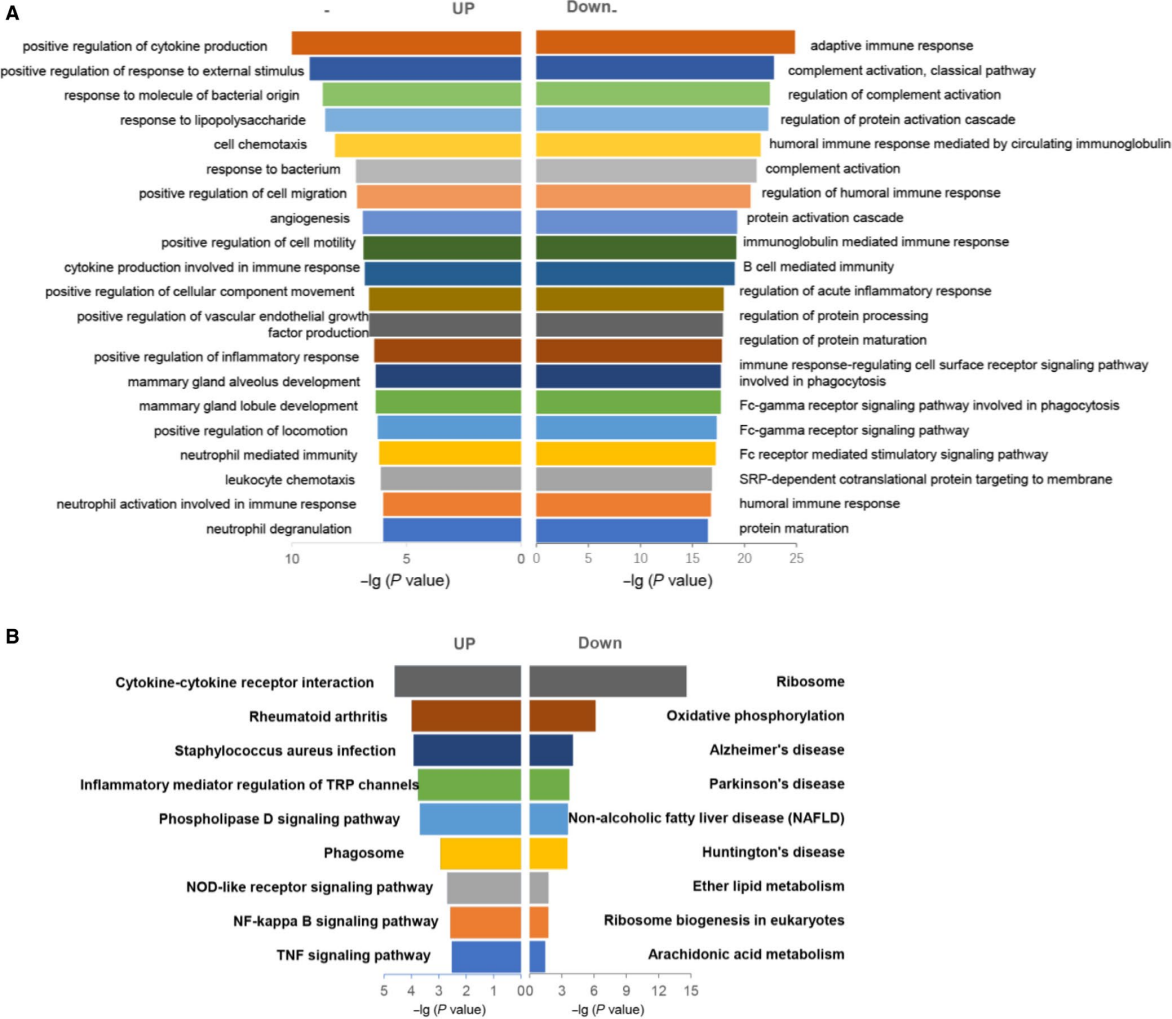
Gene ontology (GO) analysis and KEGG pathways analysis for DEGs in CHB patients and healthy controls. A, The up‐regulated and down‐regulated genes in top GO terms enriched among differentially expressed genes (DEGs), the enrichment score (−Log10 *P*‐value) of top GO terms was based on the gene expression in CHB patients compared with healthy controls. B, KEGG analysis of differentially expressed genes between CHB patients and healthy controls

To uncover the significant biological function classification of the 1401 DEGs, gene‐set enrichment analysis (GSEA) was used. First, gene ontology (GO) enrichment analysis was subjected (Figure 2A). Both the up‐ and down‐regulated expressed genes in B cells of CHB patients were further divided into three subcategories: biological progress, cell component and molecular function. The GO analysis showed that both the up‐ and down‐regulated expression genes were predominately enriched in biological process categories, and most of them were associated with immune process (Figure 2A). Many of DEGs were enriched in positive regulation of cytokine production, positive regulation of cell communication and positive regulation of signalling. Nevertheless, several signalling pathways of adaptive immune process were down‐regulated, including adaptive immune process, complement activation (classical pathway) and regulation of complement activation. In the sub‐category of molecular function, cytokine activity and cytokine binding were up‐regulated, while the signalling pathway of antigen binding was remarkably down‐regulated in GO analysis. Finally, in the cellular subcategory, the GO terms were mainly related to nucleus; membrane and cytoplasm were also changed.

To identify the significantly enriched signalling pathways, KEGG and Reactome databases were separately employed. The top 9 enriched signalling pathways for all DEGs in KEGG were listed (Figure 2B). The KEGG pathways were selected based on the threshold of a *P* value < .05. The significant pathways of up‐regulated DEGs that were mainly enriched included cytokine‐cytokine receptor interaction, rheumatoid arthritis, inflammatory mediator regulation of TRP channels, NOD‐like receptor signalling pathway, NF‐κB signalling pathway and TNF signalling pathway. On the other hand, the significant down‐regulated pathways of DEGs were enriched in ribosome, oxidative phosphorylation, non‐alcoholic fatty liver disease and ether lipid metabolism.

Additional pathway enrichment analysis was also conducted using Reactome database (Table S2), which revealed mitochondrial dysfunction as a major defect in B cells from CHB patients, ranging from mitochondrial translation initiation to termination, oxidative phosphorylation, as well while respiratory electron transport. Besides, signalling pathways regarding to p53‐dependent DNA damage response, p53 stabilization and DNA synthesis were also enriched.

### 3.3 3.3Validation of B cell hyperactivation‐related DEGs among CHB patients and healthy controls

**Figure 3:**
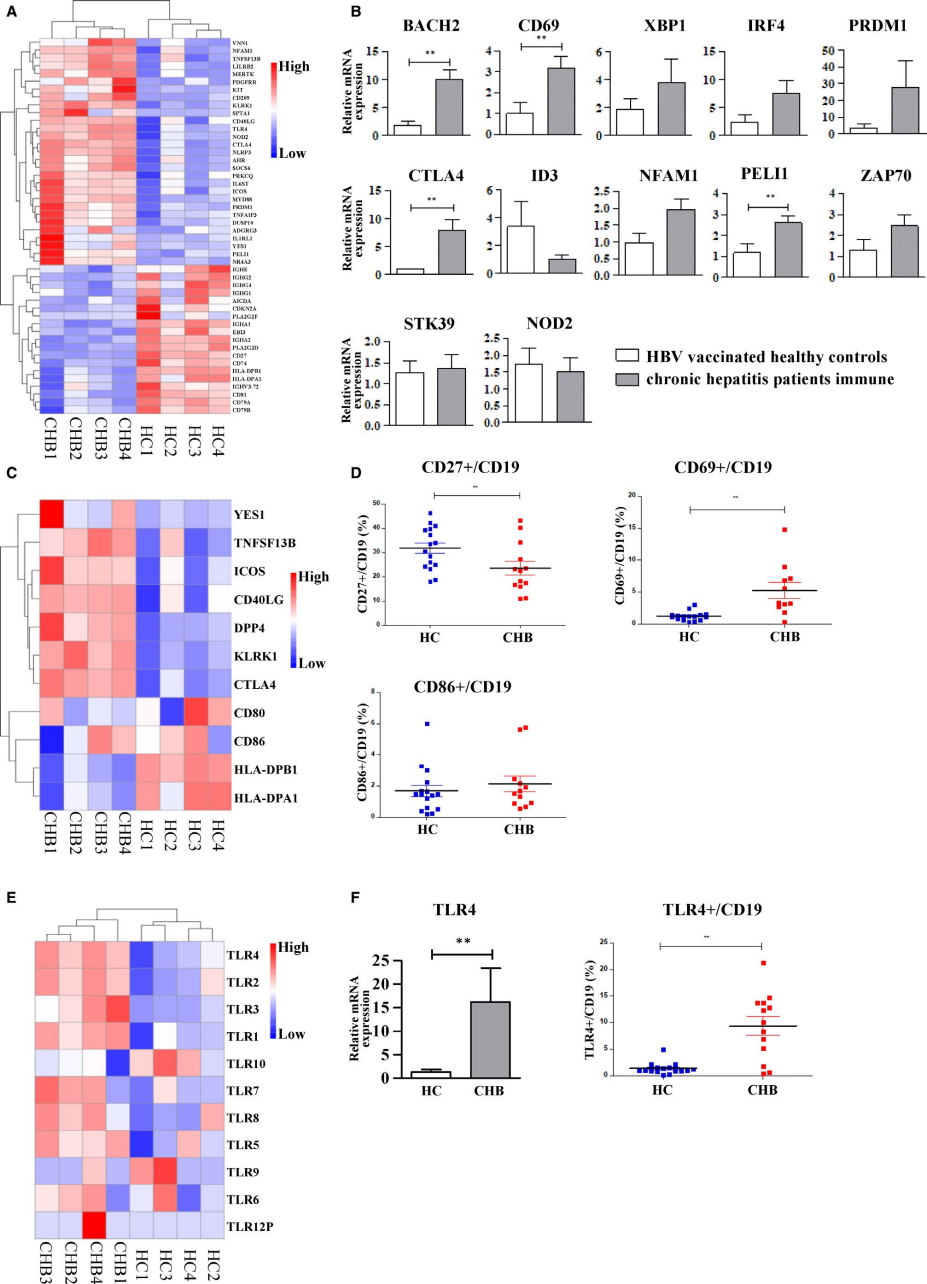
Analysis of B cell expansion and activation‐related DEGs between CHB patients and healthy controls. A, Heatmap and clustering analysis of genes related to B cell proliferation and activation. B, Validation of B cell proliferation and activation‐related differentially expressed genes (DEGs) by qRT‐PCR. C, Heatmap and clustering analysis of genes related to costimulatory receptors. D, Detection of the expression of CD69, CD27 and CD86 on B cells in CHB patients and healthy controls by flow cytometry. E, Heatmap and clustering analysis of TLR expression genes. F, The expression of TLR4 on B cells between CHB patients and healthy controls by qRT‐PCR and flow cytometry

We and others previously reported that B cells were hyperactivation during chronic B virus infection; therefore, we focused on the analysis of genes related to B cell proliferation, activation and function. Of note, as shown in the heatmap (Figure 3A), the gene expression profiles of B cell are also clustered into two distinct clusters, CHB group and HC group, respectively. The genes that promote cell proliferation and activation were significantly up‐regulated in CHB patients, including *prdm1*, *peli1*, *nfam1*, *nod2*, *ctla4*, *myd88*, *cd69*, *2bach2* and *zap70*, while the genes such as *id3*, *cd27*, *cd79a and cd79b* were down‐regulated in CHB patients. In order to verify the data obtained by RNA‐Seq analysis, the expression of 13 DEGs was further quantified by qRT‐PCR in other CHB patients and HC subjects (Table S3). Consistent with our RNA‐Seq results, *bach2*, *irf4*, *cd69* and *peli1* were significantly up‐regulated in CHB patients (Figure 3B).

It was shown that costimulatory receptors could either induce B cell proliferation and survival or regulate antibody production,^14^ but little is known about the expression of costimulatory receptors of B cells in CHB patients. As shown in our heatmap in Figure 3C, *icos*, *ctla4*, *dpp4*, *klrk1* and *cd40lg* were up‐regulated, while *hla‐dpa1* and *hla‐dpb1* were down‐regulated in patients, but without any significant difference. The expression of CD69, CD27 and CD86 on B cells from CHB patients and healthy controls was also detected by flow cytometry. In the freshly isolated PBMC from immune‐activated CHB patients, the frequency of CD27^+^/CD19^+^ B cells was significantly declined (Figure 3D), compared with HC subjects. Furthermore, the frequency of CD69^+^/CD19^+^ B cells was specially elevated in immune‐activated CHB patients than that in HC subjects (Figure 3D), while costimulatory molecule CD86 was comparable between two groups. Therefore, the above results were also consistent with the data obtained by HTS analysis and qRT‐PCR.

### 3.4 TLR4 is significantly up‐regulated in B cells from CHB patients

TLR signalling by nucleic acids is able to induce the most robust B cell activation.^15^ Thus, we comprehensively analysed TLR expression represented by heatmap (Figure 3E). Indeed, *tlr4*, *tlr2*, *tlr3* and *tlr1* were overexpressed in B cells from CHB patients. Among all TLRs tested, TLR4 was the most significantly up‐regulated, meanwhile their downstream NF‐κB pathway was significantly enriched. We further validated these observations by analysing TLR4 expression of B cells. Consistent with our RNA‐Seq results, flow cytometry analysis showed the percentage of TLR4^ +^ B cells in CHB patients was significantly higher than the healthy controls, and the mRNA level of TLR4 was also significantly up‐regulated revealed by qRT‐PCR. Our results 3F) suggested that TLR4 was remarkably up‐regulated in B cells of CHB patients.

### 3.5 TLR4 is essential for B cell hyperactivation in HBV‐carrier mouse model

To address the role of TLR4 in B cell abnormal activation in CHB patients, we utilized HBV‐carrier mouse model developed by hydrodynamic injection of the pAAV/HBV1.2 plasmid into wild‐type C57BL/10 (WT) mice and C57BL/10 TLR4^−/−^ (TLR4‐KO) mice. Similar to previous reports,^13^ serum HBsAg, HBeAg and HBV‐DNA could be detected 1 day after injection and lasted until the fourth week. Serum alanine amino transaminase activity (ALT) and aspartate aminotransferase (AST) were increased on day 1 and remained high levels thereafter.

**Figure 4:**
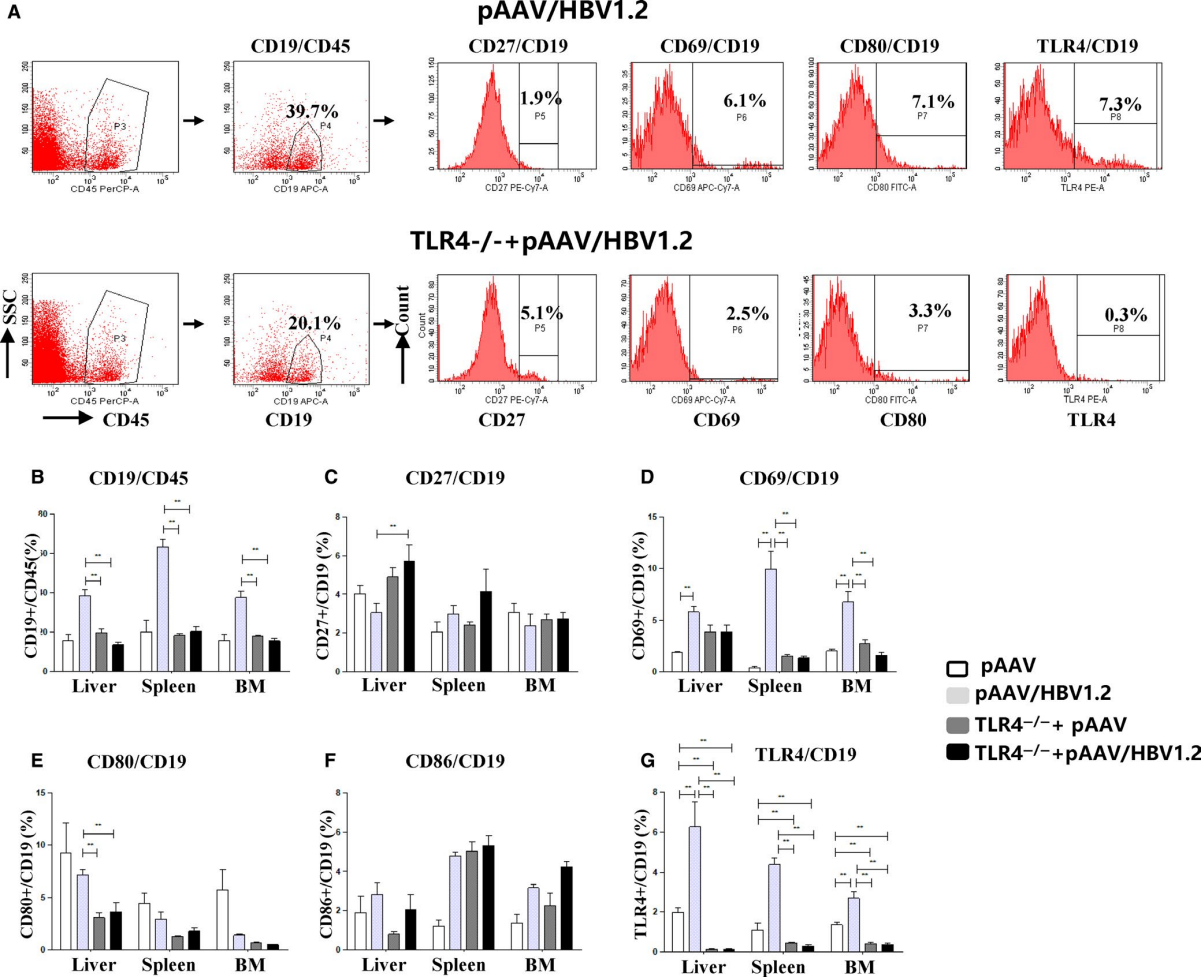
Expression of B cell proliferation and activation‐related key molecules in HBV‐carrier mouse model. The HBV‐carrier mouse model is established by hydrodynamic injection of the pAAV/HBV1.2 plasmid into wild‐type C57BL/10 (WT) mice and C57BL/10 TLR4^−/−^ (TLR4‐KO) mice. The percentage of total B cells in CD45^+^ lymphocyte was detected by flow cytometry; the expression of CD27, CD69, CD80, CD86 and TLR4 on CD19^+^ B cells was detected by flow cytometry in liver, spleen and bone marrow after pAAV/HBV1.2 injection

We first examined the number and activation status of B cells in mice by flow cytometric analysis (Figure 4A). Considering that HDI of DNA plasmid might influence the B cell status, mice received HDI of pAAV/control plasmid were used as controls. We found that the percentage of total B cells was significantly increased in liver, spleen and bone marrow after pAAV/HBV1.2 injection (Figure 4B), whereas the CD27^+^ memory B cells had no significant change (Figure 4C). Also, the activation marker, CD69, on B cells from livers and bone marrow was increased in HBV‐carrier mice (Figure 4D), whereas the expression of costimulatory molecules CD80 and CD86 on B cells from the liver, spleen and bone marrow of HBV‐carrier mice was not significantly changed (Figure 4E,F). Further, TLR4^+^ B cells were significantly increased in both liver and bone marrow of HBV‐carrier mice (Figure 4G). These data indicated that HBV is able to elicit the expansion and B cell activation in the HBV‐carrier mouse model.

To further verify the role of TLR4 in B cell activation during HBV, the percentage of B cells, memory B cells and activated B cells were also determined. Here, we found that the ablation of TLR4 failed to induce increment of B cells and activation of B cells (CD69^ +^ B cells and CD80^+^ B cells) in HBV‐carrier TLR4 KO mice, compared to HBV‐carrier TLR4 KO control mice (TLR4^−/−^/pAAV). Furthermore, the levels of serum IgG in HBV‐carrier TLR4 KO mice were significantly lower than that in HBV‐carrier WT mice, suggesting that TLR4 is essential for B cell activation and antibody production.

### 3.6 HBV induces activation and expansion of B cells through activation of TLR4‐MyD88‐NF‐κB pathway

**Figure 5:**
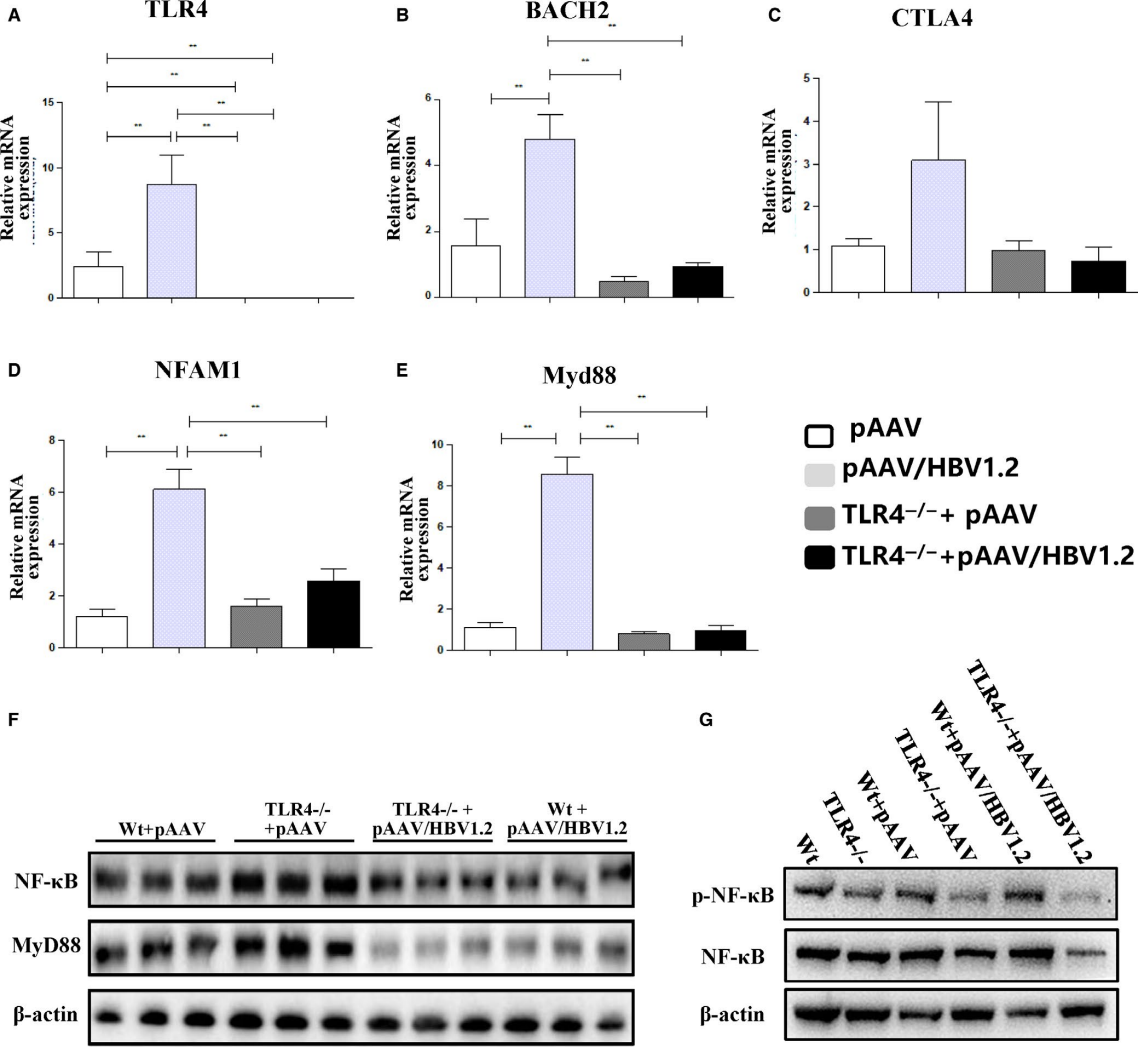
The activation of TLR4‐MyD88‐NF‐κB signalling pathway in HBV‐carrier mouse model. A‐E, Gene expression of tlr4, bach2, ctla4, nfam1 and myd88 in splenic B cells from wild‐type C57BL/10 (WT) mice and C57BL/10 TLR4^−/−^ (TLR4‐KO) mice after pAAV/HBV1.2 injection. F, Western blot analysis of protein expression of MyD88 and NF‐κB from total B cells of liver, spleen and bone marrow from HBV‐carrier mice. G, Western blot analysis of protein expression of phosphorylated NF‐κB in HBV‐carrier mice

Our RNA‐Seq data identified that *bach2*, *nfam*, *tlr4*, *myd88* and *ctla4* were up‐regulated in B cells of CHB patients, and thus, we examined the gene expression of these genes by qRT‐PCR from WT HBV‐carrier mice (Figure 5). We found that gene expression of TLR4, BACH2, NFAM1 and Myd88 was significantly augmented in splenic B cells from wild‐type HBV‐carrier mice. Further, the expression of BACH2, NFAM1 and Myd88 was substantially reduced in B cells in TLR4‐KO HBV‐carrier mice, suggesting that TLR4 can up‐regulate the expression of Myd88, BACH2 and NFAM1 triggered by HBV.

It remains to be determined that TLR4 could activate cells through its signalling adaptor MyD88 and downstream pro‐inflammatory transcription factor NF‐κB, upon the stimulation of HBV. Western blot analysis revealed that the protein expression of MyD88 and NF‐κB in HBV‐carrier mice was elevated compared with control mice (Figure 5F), suggesting TLR4/MyD88/NF‐κB pathway was activated in HBV‐carrier mice. Consistently, upon HBV stimulation, the absence of TLR4 in B cells failed to induce increased expression of MyD88 and NF‐κB. We further analysed the level of phosphorylated NF‐κB in HBV‐carrier mice. Upon stimulation of HBV, the phosphorylated NF‐κB was elevated compared with control mice, and the level of phosphorylated NF‐κB was decreased by the deletion of TLR4 in B cells (Figure 5G). These data demonstrate that TLR4 is required for HBV to activate the downstream MyD88/NF‐κB pathway in B cells, ultimately contributing to aberrant increment and activation of B cells in HBV‐carrier mice.

## 4 DISCUSSION

Accumulating clinical evidence supports that effective adaptive immune responses are essential for the clinical clearance of HBV, which drives us to understand the comprehensive gene expression signature of impaired B cells during chronic HBV infection. Here, we identified a profound and wide‐ranging cellular perturbation centred on inflammation and immune responses, which are known to negatively modulate B cell–specific activities, such as B cell activation, signalling and effector function. We validated the expression profile of genes in B cells from CHB patients by qPCR, flow cytometry and Western blot. Of note, TLR4 and downstream MyD88/NF‐κB signalling pathway were overexpressed in B cells from CHB patients. We also confirmed our observation of B cell hyperactivation in HBV‐carrier mice, while TLR4 is required for B cell hyperactivation and proliferation through the activation of MyD88/NF‐κB signalling pathway in our animal model.

In the present study, to picture the perturbed genetic profile of B cells from CHB patients, we have identified a total of 1401 genes differentially expressed between CHB and healthy control group, among which 778 genes were up‐regulated, whereas 623 genes were down‐regulated. First, we found that hyperactive processes were enriched in inflammatory response, including cytokine production, response to external stimulus, cell motility and angiogenesis. Meanwhile, a global down‐regulation of gene expression in antigen‐specific immune response was observed, including antigen binding, adaptive immune response, complement activation and immunoglobulin‐mediated immune response. These results indicated there was disturbed humoral immunity in CHB patients, in which hyperactivation of B cells was revealed but without HBV‐specific B cell responses. This is also in line with our clinical observation from CHB patients who had elevated total IgG responses whereas undetectable anti‐HBsAg antibody responses.^7^, ^10^

Our study also highlights the importance of TLR4 in B cell hyperactivation during CHB disease progression. Interestingly, B cells are a unique population of immune cells with both expressions of antigen‐specific B cell receptor (BCR) and TLR. Dual engagement of BCR and TLR is able to directly link cell‐intrinsic innate and adaptive immune response, precisely modulating B cell function. Indeed, TLRs specifically recognize conserved molecular patterns and nucleic acids as part of innate immune response, which serve as a vital role in activating the innate immune system. However, improper TLR activity might lead to hyperactive inflammation and autoimmunity.^16^ For example, peripheral B cells from patients with inflammatory bowel disease had elevated expression of TLRs.^17^ Further, TLR polymorphisms are found to be linked with the inflammatory disease such as Crohn's disease.^18^ Additionally, TLR activation such as TLR4 is linked to angiogenesis, tumour proliferation and immune evasion.^19^, ^20^ Therefore, TLRs have been identified as potential therapeutic targets in the context of chronic inflammatory diseases.^21^ We also examined various TLR expression profile of B cells in CHB patients. A panel of TLRs including TLR4, TLR2, TLR3 and TLR1 was up‐regulated in B cells among CHB patients, and TLR4 was the most significantly elevated. Such TLR activation might contribute greatly to B cell hyperactivation in CHB patients.

In B cells, TLR4 signalling through two distinct pathways, one is via the BCR leading to the activation of SYK, ERK and AKT, and the other is through MyD88 leading to the activation of NF‐κB.^22^ For example, TLR4‐MyD88‐NF‐κB signalling in B1a cells, a subset of innate‐like B cells, was shown to exert an atheroprotective effect that not only decreased lesion apoptotic cells, but also reduced T cell–augmented TGF‐β1 expression accompanied by reduced the levels of inflammatory cytokines TNF‐α, IL‐β1 and IL‐18.^23^ Interestingly, the ablation of TLR4 signals does not have any impact for B cell development and survival.^24^ In our study, TLR4 is required for B cell activation and increment in HBV‐carrier mice via the activation of downstream MyD88/NF‐κB signalling, which might contribute to hyperactivation of B cells observed in CHB patients.

TLR signalling has also been identified as a critical link between oxidative stress and inflammation.^25^ Downstream molecular pathways of TLR4 activation in the heart showed a pro‐oxidative and pro‐inflammatory state via NF‐κB activation, cytokine, and reactive oxygen species (ROS) production.^26^ Interestingly, Reactome database analysis from our study also confirmed that extensive mitochondrial defect as a major abnormality in B cells from CHB patients, ranging from mitochondrial translation initiation to termination, oxidative phosphorylation, as well as respiratory electron transport. It will be interesting to determine whether TLR4 activation is fully responsible for mitochondrial dysfunction in B cells from CHB patients. Nonetheless, correcting mitochondrial dysfunction recently has been identified as a promising target to improve exhausted CD8^+^ T cell response in CHB patients.^27^ The activation of MyD88 signalling in B cells might exert an inhibitory effect on the protective immune response. For example, during *Salmonella typhimurium* infection, selective deficiency of MyD88 in B cells improved control of bacterial replication and prolonged survival of infected mice. Further analysis revealed that MyD88 signalling in B cells suppressed the protective immunity including neutrophils, natural killer cells and inflammatory T cells, mediated by interleukin‐10 (IL‐10).^28^ Our previous work also showed that elevated levels of IL‐10 were observed, and higher frequency of B regulatory cells was identified from CHB patients.^10^ It is therefore plausible that the activation of MyD88 signalling leads to B cells secreting IL‐10, result in suppressed immune response from T cells and NK cells in CHB patients.^29^ It will be intriguing to further dissect the immune interaction network between TLR4/MyD88‐activated B cells and other immune cells especially T cells and NK cells.

Bach2 is a critical transcriptional repressor that is required for the formation of the germinal centre (GC) reactions,^30^ including class switch recombination and somatic hypermutation of Ig genes in B cells.^31^, ^32^ TLR4 signalling is able to effectively stimulate B cell maturation both in vivo and in vitro.^33^ We observed an increased expression of bach2 in B cells from HBV chronic‐infected patients compared with those healthy controls. Consistently, the level of Bach2 was decreased after depletion of TLR4 in our mouse model. Currently, it is not fully convinced to speculate that TLR4 was able to regulate Bach2 during HBV infection. Moreover, although we identified an increased level of CD27 on B cells from both WT and TLR4−/− mice after 10 days after the injection of pAAV/HBV1.2, it is not sufficient to exam the role of TLR4 in the control of memory B cell differentiation in HBV infection during such a short period of time.

In summary, TLR4 induced MyD88/NF‐κB downstream activation in B cells triggered by HBV, which provides an explanation for B cell hyperactivation observed from CHB patients. Our study also suggested that TLR4 might be used as a target for resolution of B cell hyperactivation during chronic HBV infection.

## CONFLICT OF INTEREST

The authors confirm that there are no conflicts of interest.

## AUTHOR CONTRIBUTIONS

CW and YL (Yong Liu) designed the study. YL (Yang Li), SY, YC, XT and YL (Yong Liu) performed the experiment sand analysed the data. QZ, RH, BJ, WJ, KY and JW help with some experiments. YL (Yang Li), SY, YL (Yong Liu) and CW wrote and revised the manuscript. All authors read and approved the final manuscript.

## Supporting information

Table S1

Table S2

Table S3

## Data Availability

The data sets and supporting materials generated and/or analysed during the current study are available from the corresponding author on reasonable request.
