# Supplementary figures and images for: Hepatitis B virus‐induced hyperactivation of B cells in chronic hepatitis B patients via TLR4
Source: J Cell Mol Med. 2020 May 11;24(11):6096–106. doi: 10.1111/jcmm.15202 (PMC7294113; doi:10.1111/jcmm.15202)

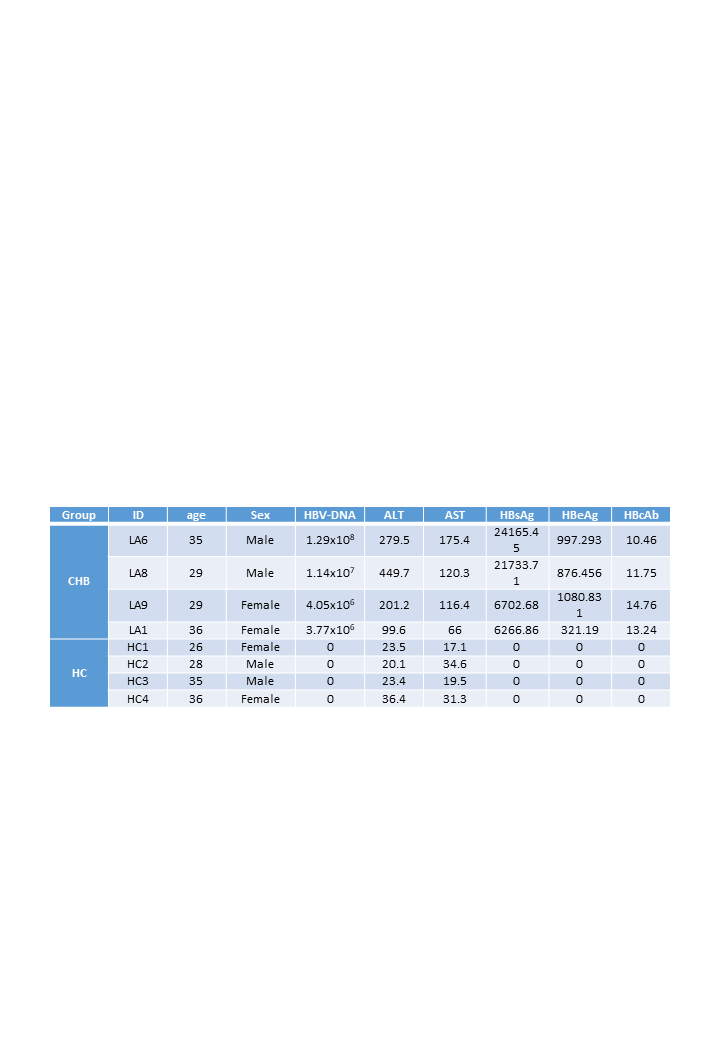

Supplement: Supplementary file 1 — Table S1 [file JCMM-24-6096-s001.TIF]

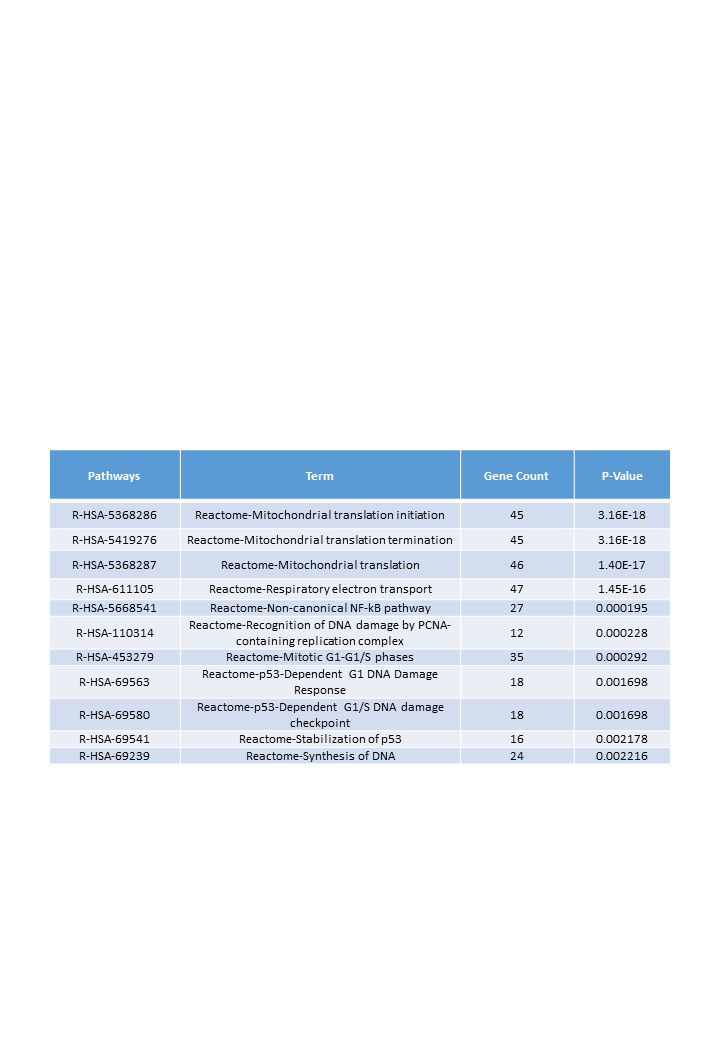

Supplement: Supplementary file 2 — Table S2 [file JCMM-24-6096-s002.TIF]

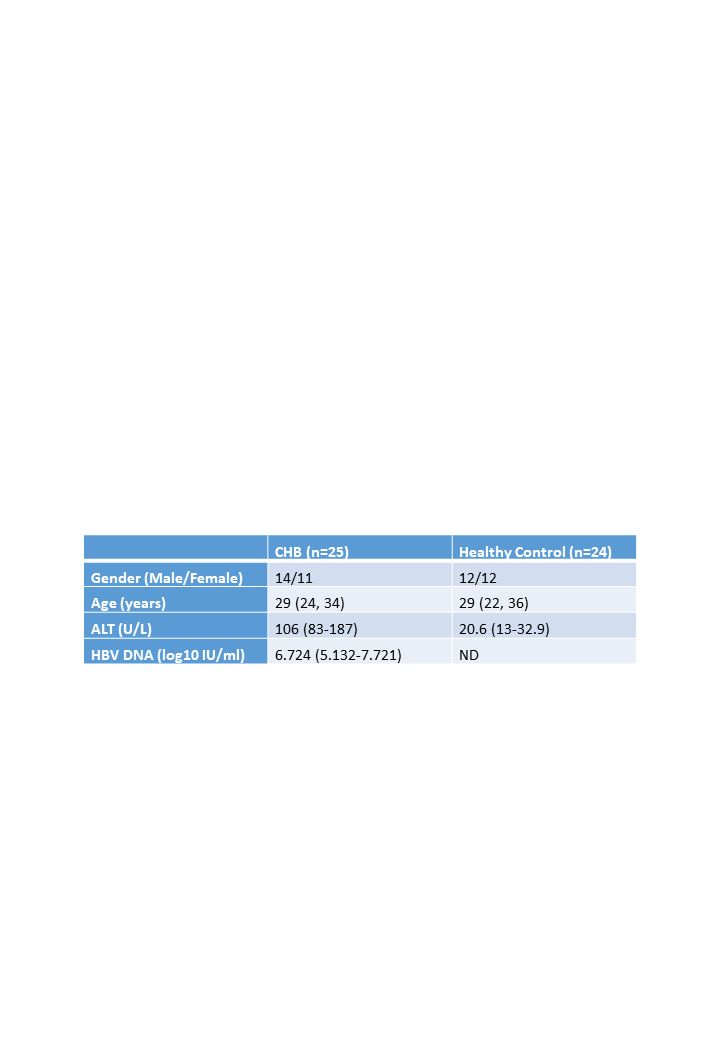

Supplement: Supplementary file 3 — Table S3 [file JCMM-24-6096-s003.TIF]
